# Modeling Networks of Coupled Enzymatic Reactions Using the Total Quasi-Steady State Approximation

**DOI:** 10.1371/journal.pcbi.0030045

**Published:** 2007-03-16

**Authors:** Andrea Ciliberto, Fabrizio Capuani, John J Tyson

**Affiliations:** 1 The FIRC Institute for Molecular Oncology, Milan, Italy; 2 Department of Biological Sciences, Virginia Polytechnic Institute and State University, Blacksburg, Virginia, United States of America; Utrecht University, The Netherlands

## Abstract

In metabolic networks, metabolites are usually present in great excess over the enzymes that catalyze their interconversion, and describing the rates of these reactions by using the Michaelis–Menten rate law is perfectly valid. This rate law assumes that the concentration of enzyme–substrate complex (*C*) is much less than the free substrate concentration (*S*_0_). However, in protein interaction networks, the enzymes and substrates are all proteins in comparable concentrations, and neglecting *C* with respect to *S*_0_ is not valid. Borghans, DeBoer, and Segel developed an alternative description of enzyme kinetics that is valid when *C* is comparable to *S*_0_. We extend this description, which Borghans et al. call the total quasi-steady state approximation, to networks of coupled enzymatic reactions. First, we analyze an isolated Goldbeter–Koshland switch when enzymes and substrates are present in comparable concentrations. Then, on the basis of a real example of the molecular network governing cell cycle progression, we couple two and three Goldbeter–Koshland switches together to study the effects of feedback in networks of protein kinases and phosphatases. Our analysis shows that the total quasi-steady state approximation provides an excellent kinetic formalism for protein interaction networks, because (1) it unveils the modular structure of the enzymatic reactions, (2) it suggests a simple algorithm to formulate correct kinetic equations, and (3) contrary to classical Michaelis–Menten kinetics, it succeeds in faithfully reproducing the dynamics of the network both qualitatively and quantitatively.

## Author Summary

The physiological responses of a cell to its environment are controlled by gene–protein interaction networks of great complexity. To understand how information is processed in these networks requires accurate mathematical models of the dynamical behavior of large sets of coupled chemical reactions. To avoid producing large and hardly manageable models, such reaction networks are often simplified using phenomenological reaction rate laws, such as the Michaelis–Menten rate law for an enzyme-catalyzed reaction. We show that, in regulatory networks where proteins swap places as enzymes and substrates, such simplifications must be carried out with care, keeping track of enzyme–substrate complexes. The risk is to provide a simplified description of the molecular networks that at best is correct for the long-term behavior but fails to represent the short-term dynamics of the real network. To avoid such a possibility, we suggest using an alternative approach called the total quasi-steady state approximation. We apply this alternative formalism to a model of the network controlling the entry into mitosis in the eukaryotic cell cycle, composed of three coupled protein modification cycles. Whereas the classical Michaelis–Menten formalism fails to represent the dynamics of this network correctly, the one we propose captures the behavior with economy and accuracy.

## Introduction

A major goal of molecular systems biology is to build, simulate, and analyze mathematical models of complex molecular regulatory systems comprising genes, proteins, and metabolites [1–3]. The bottom-up approach to this problem is to propose a hypothetical network of biochemical reactions among the component species, to formulate a set of dynamical equations (e.g., differential equations) that describe the temporal and spatial evolution of the network, to solve the equations numerically, and then to compare the model's behavior to that of living cells. The systems biologist should also analyze the generic properties of the model, as far as possible, to better understand the underlying molecular basis of cell physiology.

In principle, the governing equations for any chemical reaction network can be formulated by the law of mass action [4]; i.e., the rate of an elementary reaction A + B → … is proportional to the product of the concentrations of reactants, rate = *k* [A] [B]. (In our notation, A and B (roman) are the names of chemical species. *A* and *B* (italic) are numbers referring to the concentrations of the particular chemical species. [A] and [B] are alternative ways to express the concentrations of chemical species.) This formulation leads to many differential equations (one for every chemical species in the network, including all intermediary complexes formed, however transiently) with many separate terms on the right-hand sides (one for every reaction in which the species participates). Some of these reactions are very fast, some are very slow, and some are in between. The large differences of timescales in the network (typically many orders of magnitude) create huge difficulties for simulating the temporal evolution of the network and for understanding the basic principles of its operation.

To sidestep these problems, theoreticians often use the quasi-steady state approximation (QSSA) to eliminate the fastest (and the slowest) variables in the system of differential equations

*d*[A]/*dt* = ɛ^−1^ *f*([A],[B],[C],…)    fast
*d*[B]/*dt* = *g*([A],[B],[C],…)      intermediate
*d*[C]/*dt* = ɛ *h*([A],[B],[C],…)     slow
0 < ɛ ≪ 1.

For slow variables, the process is easy: *d*[C]/*dt* ≈ 0, [C] ≈ *C*_T_ = constant. For fast variables, it is more subtle: *d*[A]/*dt* = ɛ^−1^ *f*([A],[B],[C],…); i.e., [A] changes very rapidly until *f*([A],[B],[C],…) ≈ 0. The QSSA involves solving the algebraic equation *f*([A],[B],[C],…) = 0 for [A] = A([B],*C*_T_,…). We are left with differential equations for the medium timescale variables only,

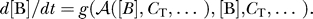



The classic example of such timescale analysis is the Michaelis–Menten (MM) theory of an enzyme-catalyzed reaction, E + S ↔ C → E + P [5]. (In our notation, rate constants are denoted *k*_1_ for binding of enzyme and substrate, *k*_–1_ for dissociation of the complex, and *k*_2_ for the catalytic reaction. We often use a letter other than *k* for these rate constants; the letter being chosen to represent the relevant enzyme in a reaction network, e.g., *d*_1_, *d*_–1_, and *d*_2_ for enzyme D; see below.) The total enzyme concentration is a slow variable (*E* + *C* = *E*_T_ = constant), the substrate concentration is the intermediate variable (*S*(*t*) changes on the timescale of interest), and the concentration of the enzyme–substrate complex, *C*, is the fast variable:

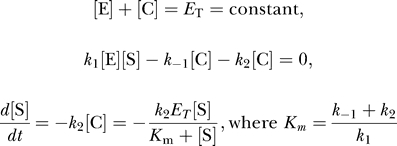



The condition for this QSSA to be valid is *E*_T_ ≪ *S*_0_ + *K*_m_, where *S*_0_ = [S](0) = initial substrate concentration.

The same sort of analysis can be carried out for networks of enzyme-catalyzed reactions, but modelers sometimes avoid the hard work of separating timescales and simply use the MM rate law to describe enzyme-catalyzed reactions in their differential equations. For protein interaction networks (PINs), such use of MM kinetics is unjustified because the enzymes have multiple substrates, the substrates are acted upon by multiple enzymes, and (worst of all) enzymes and substrates often swap roles (for example, see [6]). For instance, two kinases may phosphorylate each other, in which case it cannot possibly be true that *E*_T_ ≪ *S*_0_ + *K*_m_ for both reactions simultaneously. In this report, we show how to formulate the QSSA properly for PINs, and we address some problems in previously published models.

### The Total QSSA for Enzyme-Catalyzed Reactions

Our approach to PINs relies on a modified QSSA introduced by Borghans, DeBoer, and Segel in 1996 [7]. They proposed that, for conditions when *E*_T_ and *S*_0_ are comparable numbers, the proper intermediate timescale variable is *Ŝ*(*t*) = *S*(*t*) + *C*(*t*). In terms of this variable, the governing equations are

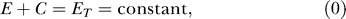


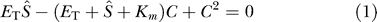


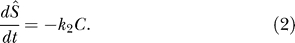



Borghans, DeBoer, and Segel called this the total QSSA (tQSSA). A sufficient condition for the uniform validity of the tQSSA was derived by Tzafriri [8]:

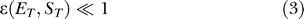
where *S*_T_ = *S*(0) + *C*(0), *ɛ*(*E*_T_,*S*_T_) = (*k*_2_/2*k*_1_ *S*_T_) · *f*(*r*(*E*_T_,*S*_T_)), *f*(*r*) = (1 – *r*)^−1/2^ − 1, and *r*(*E*_T_,*S*_T_) = 4*E*_T_ *S*_T_(*K*_m_ + *E*_T_ + *S*_T_)^−2^. Tzafriri showed that

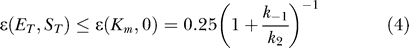
Hence, Equation 3 is satisfied if *k*_−1_ ≫ *k*_2_; i.e., the dissociation rate of the enzyme–substrate complex is much faster than the catalytic conversion of substrate into product. Notice that Equation 3 is not badly violated even if *k*_–1_ = 0; so the tQSSA is likely to be an excellent approximation for any ratio of enzyme to substrate and for any ratio of timescales.

It is of course possible, using the quadratic formula, to solve Equation 1 exactly for *C* as a function of *Ŝ* and *E*_T_ and to substitute this formula into Equation 2 for *dŜ*/*dt*. One may instead use the expression

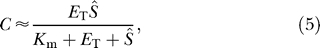
which is a good approximation so long as *C* ^2^ ≪ *E*_T_ *Ŝ*. Defining ρ = (*Ŝ* + *K*_m_)/*E*_T_, we can write the condition *C* ^2^ ≪ *E*_T_ *Ŝ* as

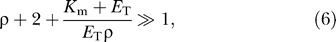
which is certainly satisfied for *ρ* ≫ 1 and *ρ* ≪ 1. At its minimum, the left-hand side of Inequality 6 is 4, so the approximate Equation 5 would seem to be quite good for any values of *E*_T_ and *S*_T_. Per Borghans, DeBoer, and Segel, we call Equation 5 the Padé approximant, and we use it whenever possible. Tzafriri [8] provides a careful discussion of the conditions for validity of the tQSSA and the more restrictive Padé approximant (which he calls the first-order tQSSA).

Recently, Pedersen et al. applied the tQSSA to the case of an enzyme converting two different substrates into products [9]. Continuing along this line, we apply the tQSSA to an isolated Goldbeter–Koshland (GK) switch [10] and then to PINs of increasing complexity.

## Results

### Analysis of the GK Module

**Figure 1:**
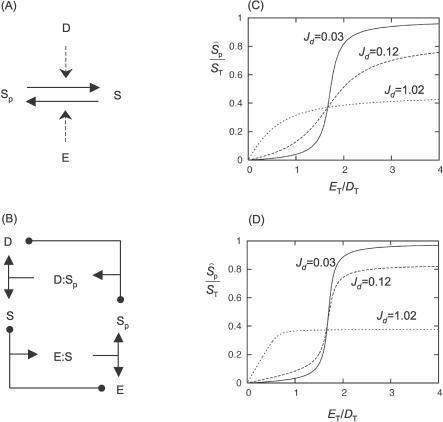
Goldbeter–Koshland Module (A) Substrate S is phosphorylated by kinase E and dephosphorylated by phosphatase D. (B) Unpacked mechanism, including enzyme–substrate (E:S) complexes. The black dots at the tips of a T-shaped arrow indicate the two molecules that come together to form a complex, pointed to by the arrowhead; the dots are meant to indicate that enzyme–substrate binding is a reversible reaction. Formation of the product (E:S → E + P) is indicated by a T with two arrowheads (pointing to E and P); absence of a dot at the foot indicates that the catalytic step is presumed to be irreversible. (C) Steady state value of *Ŝ*_p_ from Equation 8 is plotted against *E*_T_/*D*_T_ for *k*_2d_/*k*_2e_ = 1.7, *K*_me_ = *K*_md_ = 1 nM, *S*_T_ = 50 nM, and for different values of *D*_T_: 0.5 nM (solid line), 5 nM (dashed line), and 50 nM (dotted line). (D) Same as (C), but using the exact steady state equations in Table 1 instead of using the Padé approximant. For *D*_T_ = 5 nM, the exact steady state dependence is ultrasensitive, whereas the approximated dependence (C) is not.

In 1981, Goldbeter and Koshland [10] introduced the notion of an ultrasensitive switch, composed of a substrate–product pair (say, S and S_p_) that are interconverted by two enzymes (say, E and D); see Figure 1A. (Think of S_p_ as a phosphorylated form of S and of E and D as a kinase and a phosphatase, respectively.) Assuming the MM conditions, *E*_T_ ≪ *S*(0) + *K*_me_ and *D*_T_ ≪ *S*_p_(0) + *K*_md_, Goldbeter and Koshland wrote a single dynamical equation for the time evolution of the switch:

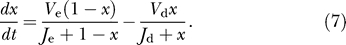
In Equation 7, *x* = *S*_p_(*t*)/*S*_T_, *S*_T_ = *S*(0) + *S*_p_(0), *J*_e_ = *K*_me_/*S*_T_, *J*_d_ = *K*_md_/*S*_T_, *V*_e_ = *k*_2e_ *E*_T_/*S*_T_, *V*_d_ = *k*_2d_ *D*_T_/*S*_T_, and the subscripts “e” and “d” refer to the kinase and phosphatase reactions, respectively. Goldbeter and Koshland showed that the steady state solution of Equation 7 is given by the GK function,

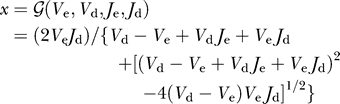



**Table 1:**
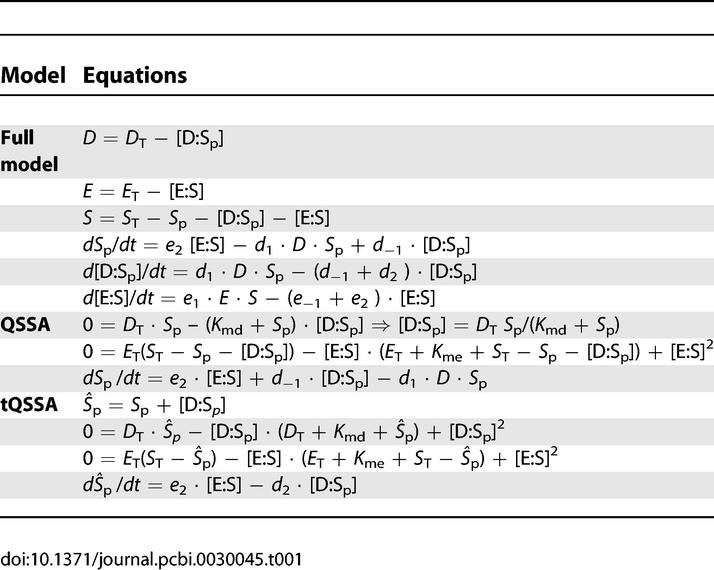
Equations for the GK Module (Figure 1)

The steady state response (*S*_p_^*^) as a function of stimulus strength (kinase level, *E*_T_) is simply

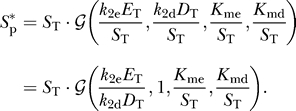
If *J*_e_ and *J*_d_ ≪ 1, then *S*_p_^*^ is a steeply sigmoidal function of *E*_T_. Goldbeter and Koshland called this signal-response curve zero-order ultrasensitivity.

Goldbeter and Koshland's analysis of phosphorylation–dephosphorylation cycles is fine for metabolic control systems, where metabolite concentrations *S*_T_ are orders of magnitude larger than enzyme concentrations, *E*_T_ and *D*_T_. But for PINs, the condition for the classical MM rate law is not valid, and we must keep track of the enzyme–substrate complexes (Figure 1B). A similar argument has recently been suggested for the mitogen-activated protein kinase pathway [11]. Re-deriving Equation 7, using the tQSSA and the Padé approximation to the algebraic equations, we find Equation 7 subject to some new definitions:

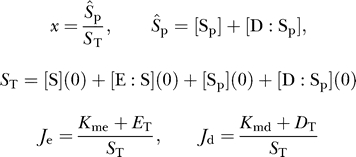



The signal–response curve,

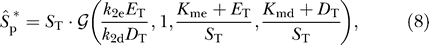
is ultrasensitive if *J*_e_ and *J*_d_ ≪ 1, but this requires that the total enzyme concentrations be small with respect to the total substrate concentration: the standard MM requirement. For PINs, we cannot expect this requirement to be satisfied, which suggests that protein phosphorylation–dephosphorylation cycles are unlikely to be ultrasensitive. In Figure 1C we plot the signal–response curve, *Ŝ*_p_/*S*_T_, as a function of *E*_T_/*D*_T_ given by Equation 8, for three different values of *D*_T_. As expected, the response function is ultrasensitive for *J*_e_ and *J*_d_ small, but ultrasensitivity is lost as *J*_e_ and *J*_d_ increase.

This conclusion about ultrasensitivity being lost in PINs, based as it is on the Padé approximant, is not reliable when enzymes and substrates are present in similar concentrations [12]. In fact, numerical calculations (Figure 1D), based on the full tQSSA equations (Table 1), show that a GK switch may still be ultrasensitive for reasonable values of *J*_e_ and *J*_d_ (see Figure 1D). Therefore, the issue of ultrasensitivity for covalent modifications in PINs should be settled using exact steady state calculations, without making the Padé approximation.

In the next section we show that the tQSSA, besides giving insights into the steady state behavior of the network, provides a good approximation of its temporal dynamics as well.

### A Numerical Example

**Figure 2:**
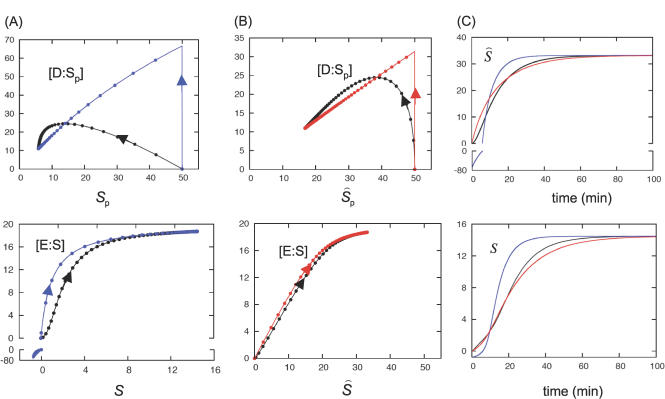
Comparison of QSSA and tQSSA for the GK Module The simulation shows the rise of *S*(*t*), starting from *S*(0) = 0, *S*_p_(0) = *S*_T_. Equations in Table 1, parameter values in Table 2. Both E and D are initially uncomplexed, *E*(0) = *E*_T_ and *D*(0) = *D*_T_. (A) Exact solution (black line), QSSA solution (blue line). The arrows indicate the direction of time, whereas the distance between two consecutive dots on the lines is 1 min. (B) Exact solution (black), tQSSA solution (red). (C) Time evolution of the same simulation, with the same color scheme. The enzyme–substrate complexes in this simulation are not negligible. In particular, at steady state, E is almost completely bound to S, whereas at the beginning of the time course, D:S_p_ accounts for roughly half of all substrate molecules.

In Table 2 (columns D and E) we assign specific values to the rate constants and total concentrations for the GK module discussed in the previous section. Our choices are not consistent with MM requirements and are only partly consistent with favorable conditions for the tQSSA. In Figure 2 we compare a numerical solution of the full set of governing differential equations with approximate solutions computed from the usual QSSA and the tQSSA (the equations are provided in Table 1 and in machine-readable form in File Collection S1). In addition to a time course (Figure 2C), we plot (Figure 2A and 2B) one of the fast variables ([E:S] or [D_p_:S_p_]) as a function of the relevant slow variable (*S* or *S*_p_ for QSSA and *Ŝ* or *Ŝ*_p_ for tQSSA), to highlight the presence of two different timescales. If the timescales are clearly separated, a sudden increase of the complex (displacement along the *y-*axis) will precede the slower conversion of substrate into product (displacement along the *x-*axis); see [7]. From the exact solution (black curves in Figures 2A and 2B) we see that, at the beginning of the reaction, S_p_ forms a complex with D, and before D:S_p_ reaches a maximum, S_p_ starts to be converted into S. As soon as S is produced, E:S is created and converted back into S_p_, with most of E forming a stable complex with S ([E:S]/*E*_T_ = 0.935 at steady state). Although the fast and slow dynamics are not perfectly separated in either QSSA or tQSSA, the timescale separation is clearly more pronounced in tQSSA (red curve in Figure 2B) than in QSSA (blue curve in Figure 2A). Time courses (Figure 2C) show that throughout the simulation tQSSA does a better job in reproducing the dynamics of the network than QSSA. Notice that at the beginning of the simulation, QSSA gives negative values to *S* and [E:S] to comply with both initial conditions and conservation relations.

**Table 2:**
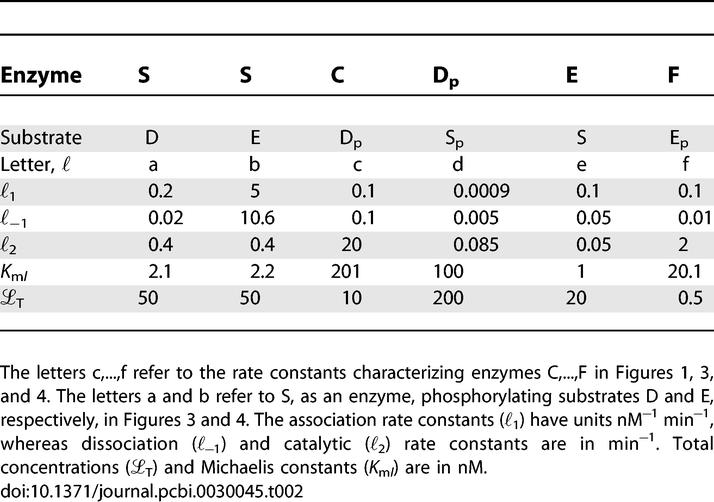
Parameter Values, ℓ_i_, for the Models: ℓ = letter (a,. . . , f) and *i* = 1, −1, 2

**Figure 3:**
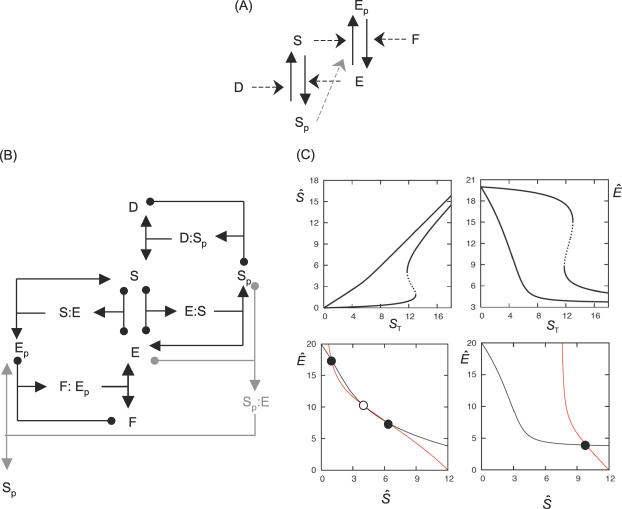
Mutual Antagonism between Two Kinases (A) A simplified diagram shows all the actors of the network: two kinases S and E, and two phosphatases D and F. In grey, the additional reaction whereby S_p_ retains some catalytic activity. (B) The unpacked diagram, keeping track of all enzyme–substrate complexes. Diagram conventions as in Figure 1. (C) Upper panels: bifurcation diagrams for the exact model, equations in Table 3, parameter values in Table 2. Solid lines represent stable steady states; dashed lines are unstable steady states. When S_p_ has no catalytic activity, the system does not show hysteresis. Hysteresis is recovered if S_p_ has some residual activity—grey lines in (A) and (B). The background activity has the following parameter values: *b*′_1_= 0.05 nM min^−1^, *b*′_−1_= 0.005 min^−1^, and *b*′_2_= 0.0001 min^−1^. (C) Lower panels: phase plane diagrams for the tQSSA model with *S*_T_=12. *Ê* nullcline (black), *Ŝ* nullcline (red), stable steady states (black dots), unstable steady state (white dot). Left: when S_p_ has some catalytic activity, the system is bistable. Right: when S_p_ has no catalytic activity, the system is monostable.

**Figure 4:**
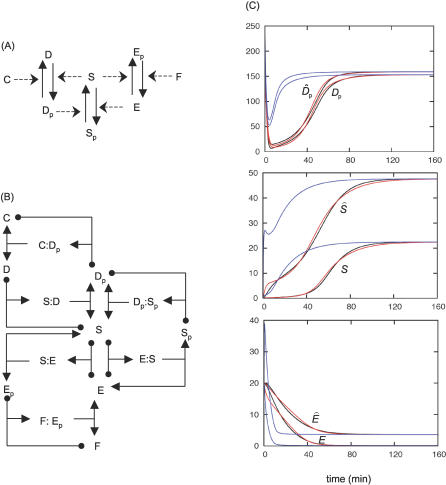
The Novak–Tyson Model for the G_2_/M Transition (A) A simplified diagram shows all the actors of the network: two kinases S and E, and three phosphatases C, D, and F. (B) The unpacked diagram, keeping track of all enzyme–substrate complexes. Diagram conventions as in Figure 1. (C) The time course of the network shown in (B); equations in Table 4, parameter values in Table 2. Initial conditions: *D*_p_ = 200 nM, *S* = 0, *E* = 20 nM, all complexes = 0. Again, QSSA (blue) performs poorly in reproducing the dynamics of the full system (black), whereas tQSSA (red) is a good approximation. Comparisons between D, S, and E and their counterparts *D2*, *Ŝ*, and *Ê* show that while the complexes forming D are not present in significant amounts, S contributes about one half of *Ŝ*, and E's contribution to *Ê* is negligible.

**Table 3:**
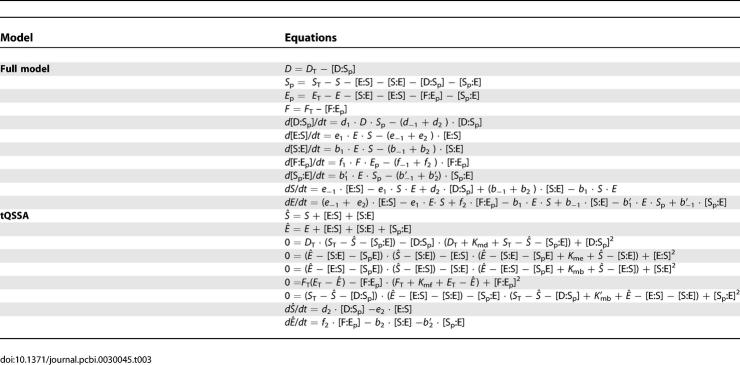
Equations for the S/E Module (Figure 3)

**Table 4:**
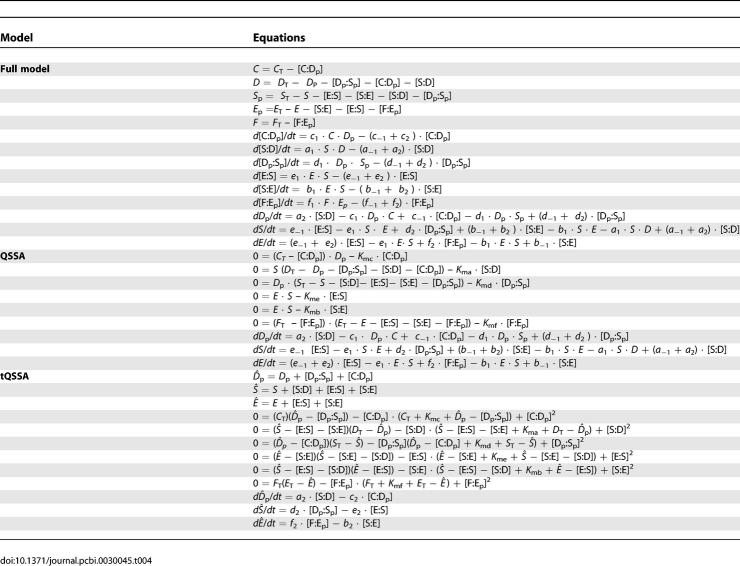
Equations for the Network of Figure 4B

### Coupled GK Modules: The Antagonism between MPF and Wee1

In this section we study the steady state behavior of a system of two coupled GK switches (Figure 3A). Building upon the previous network, we consider the possibility that E exists in phosphorylated (E_p_) and unphosphorylated (E) forms. Suppose that E_p_ is a less active form, and E → E_p_ is catalyzed by S. S and E are antagonists since they phosphorylate and inactivate each other. F is a phosphatase that converts E_p_ back to E. (In our notation for complexes, A:B, the enzyme comes first and substrate follows; for example, for the reaction whereby S phosphorylates E, the enzyme–substrate complex is denoted S:E.)

This is no arbitrary example; it describes exactly the interactions between two regulators of the G_2_-to-mitosis (G_2_/M) transition in the eukaryotic cell cycle [13]. In that case, S = MPF (M-phase promoting factor, a dimer of Cdc2 and cyclin B) and E = Wee1 (a kinase that phosphorylates and inactivates Cdc2). The parameter values that we choose for these coupled enzymatic reactions (Table 2) are taken, for the most part, from a careful study of the biochemical kinetics of these reactions in *Xenopus* egg extracts, done by Marlovits et al. [14]. Novak, Tyson, and collaborators implemented these values into a mathematical model for the G_2_/M transition during early embryonic cell cycles. The equations of the model were derived from an implicit application of the QSSA, but to our knowledge an explicit derivation has never been done: this observation prompted us to investigate the matters addressed in this paper.

The antagonism between E and S (Figure 3A) constitutes a positive feedback loop (sometimes called a double-negative feedback loop). Novak and Tyson proposed that this loop contributes to the bistability that characterizes the G_2_/M transition in the eukaryotic cell cycle [15–17]. In fact, according to their original model, the antagonism between Wee1 and MPF alone could sustain bistability. However, this conclusion was drawn from an improper application of the QSSA; hysteresis is lost once the network is unpacked to its elementary steps (Figure 3B). Even more, according to Advanced Deficiency Theory (performed with the software package CRNT [Chemical Reaction Network Toolbox] developed by Feinberg [18]), this network (Figure 3B) cannot have bistable behavior for any positive values of the kinetic rate constants. Is it possible to recover hysteresis from the antagonism between S and E?

In Novak and Tyson's original model, both S_p_ and E_p_ had some residual activity, a feature that in their model was not essential to generate hysteresis and that we have not taken into account so far. This residual activity becomes essential when the network is reduced to elementary steps: we find that bistability is recovered when we add the reactions E + S_p_ ↔ S_p_:E → E_p_ + S_p_, i.e., when S_p_ retains some limited kinase activity. (In Figure 3B the additional reactions are drawn in grey, and in Figure 3C, top panel, the signal–response curve is drawn with and without the additional reactions.) Bifurcation analysis suggests that the new reactions create a steady state where S is inhibited and E is active, which is paradoxical because the additional reactions provide an alternative path for inactivating E. The paradox is resolved when we realize that a large fraction of S is sequestered in S_p_:E complexes, thus helping E to inactivate S. Indeed, hysteresis is possible (unpublished data) with the association–dissociation reactions alone (E + S_p_ ↔ S_p_:E) without the catalysis step (S_p_:E → E_p_ + S_p_). Of course, bistability can also be restored by allowing E_p_ to phosphorylate S.

To apply the tQSSA to such networks, we first define “hat” variables to include a single free molecular species plus all the complexes in which this species appears. Defined thus, the association–dissociation reactions and all the reactions where a chemical species serve as a catalyst cancel out. Here we define

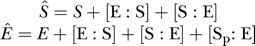
whose rates of change are given by

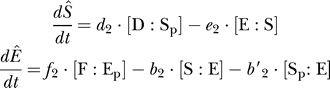



Our definition of hat variables extends what was originally proposed for a single enzymatic reaction by Borghans, DeBoer, and Segel. In terms of the hat variables, we can describe each catalytic reaction in the network with equations similar to Equations 1 and 2. For example, the rate of phosphorylation of S in Figure 3B is described by Equation 2 with *k*_2_ *C* → *e*_2_ [E:S] and with the concentration of enzyme–substrate complex given by Equation 1 with *C* = [E:S], 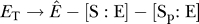 , and 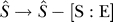 (see equations in Table 3).

Concerning this network: we perform no numerical simulations to compare tQSSA and QSSA; we will do that in the next section for a larger and biologically more significant network. Rather, we use the model reduced with tQSSA to perform phase plane analysis (Figure 3C, bottom panel), which confirms that the additional reaction strengthens the negative effect that E exerts on S, thereby bending the *Ŝ* nullcline and creating three intersection points.

### Coupled GK Modules: The G_2_/M Transition Network

In the G_2_/M network, the antagonism between Wee1 and MPF is aided by a second positive feedback loop, involving MPF and Cdc25, a phosphatase that removes the inactivating phosphate group from Cdc2 [13]. (In our notation, Wee1 is E, MPF is S, and Cdc25 is D.) Because S can phosphorylate D, and D_p_ is a more active form, S and D_p_ activate each other. Finally, we have C, an unregulated phosphatase that converts D_p_ back to D.

Altogether, the network consists of three GK modules: the first (C/D/S) controls D's phosphorylation, the second (D/S/E) affects the phosphorylation state of S, and the last (S/E/F) controls the activity of E (Figure 4A and 4B). Again, we take most of the parameter values for the additional reactions from Marlovits et al. [14]. In Table 4 we provide the governing equations for these coupled GK switches in three versions: full (no approximations), QSSA, and tQSSA.

Bifurcation analysis and CRNT show that the network is bistable even with no residual activity for S_p_ or E_p_ (unpublished data). Indeed, the positive feedback between D_p_ and S suffices, by itself, to generate hysteresis, as confirmed by CRNT. Interestingly, given the parameter values in Table 2, the network is bistable (unpublished data) in a region very similar to that determined experimentally by Sha et al. [17].

Since the model performs satisfactorily concerning its steady state behavior, we move on to compare the dynamics of both QSSA and tQSSA to the exact solution (Figure 4C). To apply tQSSA, we define the hat variables

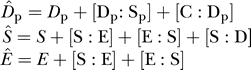
whose dynamics are described by the following equations:

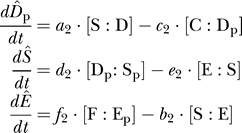
The concentrations of the enzyme–substrate intermediates are given by solving simultaneously a set of six coupled quadratic algebraic equations (see Table 4). Notice how the modularity of the network (composed of six identical MM reactions) is mirrored by the modularity of the tQSSA equations. Numerical simulations (Figure 4C) demonstrate the superiority of tQSSA (red lines) over QSSA (blue lines) in accurately capturing the exact dynamics (black lines) of this regulatory network.

**Figure 5:**
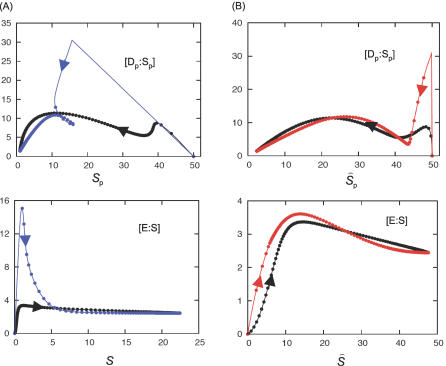
Timescale Separation in the Model of the G_2_/M Network The exact solution (black lines) is compared with the QSSA, blue lines in (A), and to the tQSSA, red lines in B. Arrows indicate the direction of time, whereas the distance between consecutive dots on the lines is 1 min. Equations in Table 4, parameter values in Table 2.

To compare further the QSSA and tQSSA with the exact solution, we plot in Figure 5 the complexes ([D_p_:S_p_] and [E:S]) as functions of the slow variables (*S*_p_ and *S* for QSSA and *Ŝ*_p_ and *Ŝ* for tQSSA). The initial hump of [D_p_:S_p_]—due to phosphorylation of D by S followed by dephosphorylation of D_p_ by C—(black curves in Figure 5A and 5B), is captured qualitatively by the tQSSA (red curve) but completely missed by the QSSA (blue curve). Similarly, the initial accumulation of [E:S] is closely approximated by the tQSSA but badly overshot by the QSSA. Both approximations capture the latter part of the time course reasonably well. The QSSA completely misses the initial stages of the simulation because it assigns negative values to *E*_p_, [F:E_p_], and *D*.

## Discussion

Coupled enzymatic reactions (e.g., interacting kinases and phosphatases) are common features of PINs. We analyze one of these networks, composed of three phosphorylation and three dephosphorylation reactions, altogether comprising 14 chemical species. The network was originally proposed by Novak and Tyson [13] to model the activation of MPF in early embryos of the frog *Xenopus*. In their original work, Novak and Tyson neglected the contribution of enzyme–substrate complexes. Using a combination of mass-action and MM kinetics, they showed that the network may have multiple steady state solutions. We have relaxed the assumptions, writing down differential equations for each species, including the enzyme–substrate complexes. Using parameters obtained indirectly from published data, we confirmed that the model exhibits bistability in the same parameter range as predicted theoretically by Novak and Tyson [13] and measured experimentally [16,17]. As for the dynamics, our simulations show that the enzyme–substrate complexes, neglected in the original model, are present in non-negligible concentrations.

The complexes play an important role because of the topology of the network. In the cell cycle model, some molecular species are at the same time enzymes and substrates of each other. If for one reaction enzyme concentration is negligible compared with substrate concentration, then the opposite must be true when the roles are exchanged, allowing for a significant fraction of the substrate to be sequestered in the complex. Similarly, when some molecules form complexes with several enzymes, even if each complex is not present in a large amount, their sum may not be negligible.

The role played by enzyme–substrate complexes in PINs could be more important than currently appreciated. In the cell cycle network, Wee1 and MPF are antagonists that phosphorylate and inhibit each other. In our simulations, the concentrations of Wee1:MPF and MPF:Wee1 are not negligible. The presence of high concentrations of such complexes can be interpreted as a second way for Wee1 to inhibit MPF, by sequestration. In this sense, Wee1 behaves as both an inhibitory kinase and a stoichiometric cyclin dependent kinase (CDK) inhibitor. In our simulation we notice that MPF:Cdc25 is also present in high concentration (40% of total MPF, 10% of total Cdc25). If confirmed, that would suggest an intrinsic way to attenuate the positive feedback loop between MPF and Cdc25. Finally, trimeric complexes might form as well (e.g., Wee1:MPF:Wee1), and such molecular species may have great effects on the qualitative behavior of PINs (Sabouri-Ghomi, Ciliberto, Novak, and Tyson, unpublished data).

Of course, one can follow the exact dynamics of a control system by solving the full system of ordinary differential equations (ODEs). However, a full description of the system comes with many equations, making any qualitative analysis difficult. A typical way to reduce the number of equations is the QSSA originally formulated for an isolated enzyme-catalyzed reaction, when substrate is in great excess over enzyme. When applying the QSSA to a network of coupled catalytic reactions, with realistic values of rate constants and total protein concentrations, we found that the reduced system of ODEs obtained by the QSSA does not faithfully reproduce the dynamics of the full system of ODEs.

The tQSSA works for a larger range of parameter values than does the QSSA; in particular, it is valid even when the enzyme is in excess compared with the substrate. Such an approximation is particularly appealing in PINs where, as mentioned above, enzymes and substrates often exchange roles. Given its appeal, we applied tQSSA to a full model of the PIN that regulates the G_2_/M transition in the cell cycle. We found by numerical simulations that the tQSSA does a good job describing coupled catalytic reactions. Moreover, applying tQSSA to PINs generates a set of differential–algebraic equations of standard format, Equations 1 and 2, suggesting that a computer algorithm may be devised whereby tQSSA is used to reduce a full set of multi-timescale ODEs to a smaller set of slow equations. The reduced set of equations may be especially useful for stochastic simulations of PINs by Gillespie's algorithm.

Summarizing, we propose that large networks of coupled enzymatic reactions should first be written in full and then reduced by applying the tQSSA. This way it will be possible to reduce the number of dynamic equations while maintaining the complexity of the network (i.e., including enzyme–substrate complexes) and simultaneously to achieve reliable approximate solutions for the transient dynamics of the network.

## Methods

All calculations have been made using XPPAUT, software developed by Ermentrout [19] and freely available online at http://www.math.pitt.edu/∼bard/xpp/xpp.html. XPPAUT solves the algebraic part of differential–algebraic equations by Newton's method, given an initial guess for the unknowns. In File Collection S1 we provide .ode files, readable by XPPAUT, for reproducing the results in this paper.

## Supporting Information

File Collection S1 .ode Files We provide .ode files, readable by XPPAUT, for reproducing the results in this paper.
(6 KB ZIP)

## Abbreviations

CRNT: Chemical Reaction Network Toolbox
GK: Goldbeter–Koshland
MM: Michaelis–Menten
MPF: M-phase promoting factor
ODE: ordinary differential equation
PIN: protein interaction network
QSSA: quasi-steady state approximation
tQSSA: total quasi-steady state approximation

## References

[pcbi-0030045-b001] Bray D (1995). Protein molecules as computational elements in living cells. Nature.

[pcbi-0030045-b002] Hartwell LH, Hopfield JJ, Leibler S, Murray AW (1999). From molecular to modular cell biology. Nature.

[pcbi-0030045-b003] Tyson JJ, Chen K, Novak B (2001). Network dynamics and cell physiology. Nat Rev Mol Cell Biol.

[pcbi-0030045-b004] Tyson JJ, Novak B, Odell GM, Chen K, Thron CD (1996). Chemical kinetic theory: Understanding cell-cycle regulation. Trends Biochem Sci.

[pcbi-0030045-b005] Segel LA (1988). On the validity of the steady state assumption of enzyme kinetics. Bull Math Biol.

[pcbi-0030045-b006] Bluthgen N, Herzel H (2003). How robust are switches in intracellular signaling cascades?. J Theor Biol.

[pcbi-0030045-b007] Borghans JA, de Boer RJ, Segel LA (1996). Extending the quasi-steady state approximation by changing variables. Bull Math Biol.

[pcbi-0030045-b008] Tzafriri AR, Edelman ER (2004). The total quasi-steady-state approximation is valid for reversible enzyme kinetics. J Theor Biol.

[pcbi-0030045-b009] Pedersen MG, Bersani AM, Bersani E (2007). The total quasi-steady-state approximation for fully competitive enzyme reactions. Bull Math Biol.

[pcbi-0030045-b010] Goldbeter A, Koshland DE (1981). An amplified sensitivity arising from covalent modification in biological systems. Proc Natl Acad Sci U S A.

[pcbi-0030045-b011] Bluthgen N, Bruggeman FJ, Legewie S, Herzel H, Westerhoff HV (2006). Effects of sequestration on signal transduction cascades. FEBS J.

[pcbi-0030045-b012] Tzafriri AR (2003). Michaelis–Menten kinetics at high enzyme concentrations. Bull Math Biol.

[pcbi-0030045-b013] Novak B, Tyson JJ (1993). Numerical analysis of a comprehensive model of M-phase control in Xenopus oocyte extracts and intact embryos. J Cell Sci.

[pcbi-0030045-b014] Marlovits G, Tyson CJ, Novak B, Tyson JJ (1998). Modeling M-phase control in Xenopus oocyte extracts: The surveillance mechanism for unreplicated DNA. Biophys Chem.

[pcbi-0030045-b015] Cross FR, Archambault V, Miller M, Klovstad M (2002). Testing a mathematical model of the yeast cell cycle. Mol Biol Cell.

[pcbi-0030045-b016] Pomerening JR, Sontag ED, Ferrell JE (2003). Building a cell cycle oscillator: Hysteresis and bistability in the activation of Cdc2. Nat Cell Biol.

[pcbi-0030045-b017] Sha W, Moore J, Chen K, Lassaletta AD, Yi CS (2003). Hysteresis drives cell-cycle transitions in Xenopus laevis egg extracts. Proc Natl Acad Sci U S A.

[pcbi-0030045-b018] Craciun G, Tang Y, Feinberg M (2006). Understanding bistability in complex enzyme-driven reaction networks. Proc Natl Acad Sci U S A.

[pcbi-0030045-b019] Ermentrout B (2002). Simulating, analyzing and animating dynamical systems: A guide to XPPAUT for researchers and students.

